# Improved gut microbiome recovery following drug therapy is linked to abundance and replication of probiotic strains

**DOI:** 10.1080/19490976.2022.2094664

**Published:** 2022-08-02

**Authors:** Jamie FitzGerald, Shriram Patel, Julia Eckenberger, Eric Guillemard, Patrick Veiga, Florent Schäfer, Jens Walter, Marcus J Claesson, Muriel Derrien

**Affiliations:** a School of Microbiology, University College Cork, Cork, Ireland; b APC Microbiome Ireland, University College Cork, Cork, Ireland; c Advanced Health & Science, Danone Nutricia Research, Palaiseau, France

**Keywords:** Antibiotics, gut microbiome recovery, fermented milk product, probiotics, replication, *L. paracasei* CNCM I-1518

## Abstract

Probiotics have been used for decades to alleviate the negative side-effects of oral antibiotics, but our mechanistic understanding on how they work is so far incomplete. Here, we performed a metagenomic analysis of the fecal microbiota in participants who underwent a 14-d *Helicobacter pylori* eradication therapy with or without consumption of a multi-strain probiotic intervention (*L. paracasei* CNCM I-1518, *L. paracasei* CNCM I-3689, *L. rhamnosus* CNCM I-3690, and four yogurt strains) in a randomized, double-blinded, controlled clinical trial. Using a strain-level analysis for detection and metagenomic determination of replication rate, ingested strains were detected and replicated transiently in fecal samples and in the gut during and following antibiotic administration. Consumption of the fermented milk product led to a significant, although modest, improvement in the recovery of microbiota composition. Stratification of participants into two groups based on the degree to which their microbiome recovered showed i) a higher fecal abundance of the probiotic *L. paracasei* and *L. rhamnosus* strains and ii) an elevated replication rate of one strain (*L. paracasei* CNCMI-1518) in the recovery group. Collectively, our findings show a small but measurable benefit of a fermented milk product on microbiome recovery after antibiotics, which was linked to the detection and replication of specific probiotic strains. Such functional insight can form the basis for the development of probiotic-based intervention aimed to protect gut microbiome from drug treatments.

## Introduction

Antibiotics are essential to treat bacterial infections and are life-saving medications. However, their use has been associated with the alteration in the human gut microbiome that may lead to short-term effects such as Antibiotic Associated Diarrhea (AAD), emergence of antibiotic resistance in both commensal and pathogenic intestinal bacteria, and increased susceptibility to pathogen colonization, and eventually chronic diseases in the longer term^1^. Most antibiotics at sufficient doses cause rapid alteration of gut microbiota, a decrease in richness and diversity, a bloom of pathobionts of the family *Enterobacteriaceae*, and depletion of several taxa including *Bifidobacterium* and butyrate producers (reviewed in^2^). Microbiomes tend to revert back toward the pre-treatment state once antibiotic treatment is stopped, but this process is incompletely understood, and recovery is often incomplete and personalized,^3^ resulting in bacterial species to be lost.^4^

Strategies to promote gut microbiota structural and functional recovery following exposure to antibiotics include defined approaches such as pre- and probiotics.^5–7^ Although probiotics show species-specific benefits on AAD,^8^ the reported effects on gut microbiota recovery have been inconsistent between studies, which differ substantially in design, population, and the probiotic strains and antibiotics used. Two recent studies in healthy subjects showed contradictory findings in that post-antibiotic gut microbiome recovery is either improved by a fermented milk product containing *Bifidobacterium animalis* subsp *lactis* BB-12^9^ or substantially impaired by a multi-strain product.^10^ These contradictions emphasize the limitations in our ecological understanding of the impact of probiotics, and the effects of specific strains, on microbiome recovery.

Research so far has provided limited insight into the mechanisms by which probiotics influence gut microbiome recovery after antibiotics. Most studies rely on 16S rRNA gene sequencing, which does not provide functional insight about the microbial community and reliable taxonomic resolution beyond genera. In contrast, whole metagenome sequencing (WMS) allows both the analysis of the functions encoded by the genes present in the microbiome and reliable taxonomic resolution at the level of species and subspecies.^11^ WMS further allows the specific detection of probiotic strains through single nucleotide variant calling^12^ or read mapping against reference genomes.^13^ Advanced bioinformatic approaches have further been developed to estimate the in-situ microbial growth rate.^14–17^ However, so far, few studies have used WMS to elucidate the effect of probiotic strains on microbiome recovery after antibiotics, leaving a gap in our understanding on how specific probiotic strains impact post-antibiotic microbiota assembly.

A fermented milk product containing the strain *Lacticaseibacillus paracasei* (*L. paracasei*) CNCM I-1518 was previously shown to reduce the occurrence of antibiotic-associated diarrhea (AAD) and *Clostridioides difficile* -associated diarrhea in hospitalized elderly^18,19^ and the incidence of common infectious diseases in the general population.^20^ Furthermore, we have selected two strains, *L. paracasei* CNCM I-3689 on the basis of pathobiont clearance,^21^ and *Lacticaseibacillus rhamnosus* (*L. rhamnosus*) CNCM I-3690 for immune modulation and support of intestinal barrier integrity^22,23^ . Recently, a seven-strain fermented milk product containing *L. paracasei* CNCM I-1518, *L. paracasei* CNCM I-3689, *L. rhamnosus* CNCM I-3690, and four strains of species used as classic yogurt starters (*Lactobacillus delbrueckii* subsp. *bulgaricus* (*L. bulgaricus*) and *Streptococcus salivarius* subsp. *thermophilus* (*S. thermophilus*)) was tested in a randomized, double-blind, controlled trial in 136 adults under *Helicobacter pylori*-eradication therapy (14-d amoxicillin, clarithromycin, and pantoprazole). This study showed that the fermented milk product induced a faster recovery (beta-diversity) of global gut microbiota composition characterized by 16S rRNA gene sequencing.^24^ In this study, we extended this research using WMS with the goal to gain functional and mechanistic insights into the effects of the fermented milk product and the contribution of the individual strains on the ecological and functional characteristics of gut microbiome recovery.

## Results

### Study design

**Figure 1.**
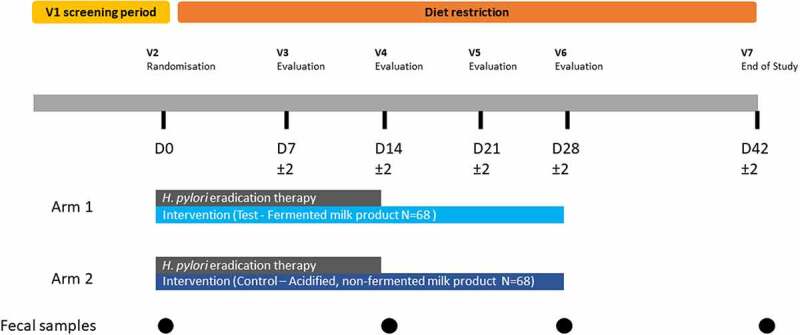
Clinical study design.

The fecal samples analyzed here were previously collected during a randomized, double-blind, controlled trial in subjects presenting infection with *Helicobacter pylori* (*Hp*) who underwent a 14-d *Hp* eradication therapy with or without consumption of a multi-strain probiotic intervention.^24^ *Hp* eradication therapy consisted of a course of two antibiotics, amoxicillin (beta-lactam) and clarithromycin (macrolide), and a proton pump inhibitor (PPI, pantoprazole). Subjects were randomized toreceive either a fermented milk product (‘Test product’) with probiotics (N = 68, ‘Test’ group) or the control, an acidified but non-fermented milk product (N = 68, ‘Control’ group), during the course of the 2-week *Hp* therapy and 2 weeks afterward (for a total of 4 weeks), as has been previously described^24^ (Figure 1). No significant difference that could impact the evaluation of product effect was observed between groups for major known confounding factors of gut microbiota such as age, sex, BMI, physical activity, dietary intake (including dietary fiber or alcohol), and smoking habits (Table S1).^24^ Additionally, the proportion of Test and Control subjects with successful eradication of *Hp* was similar between both groups (>80%) (Table S1). Fecal samples from 135 subjects (67 in Test and 68 in Control group) collected at four time points, baseline (D0), end of *Hp* eradication therapy (D14), and 2 and 4 weeks after therapy (D28, D42), were available for the current study and subjected to deep WMS. Sequencing resulted in an average of 30 million read-pairs (SD: ±27%) per sample. Comparison of D0 and D14 samples allows the analysis of the acute response to the challenge (*Hp* therapy), whereas the analysis of samples collected during the post-therapy period between D28 and D42 allows insight into microbiome recovery.

### Administered bacterial strains are transiently replicating in the gut

**Figure 2.**
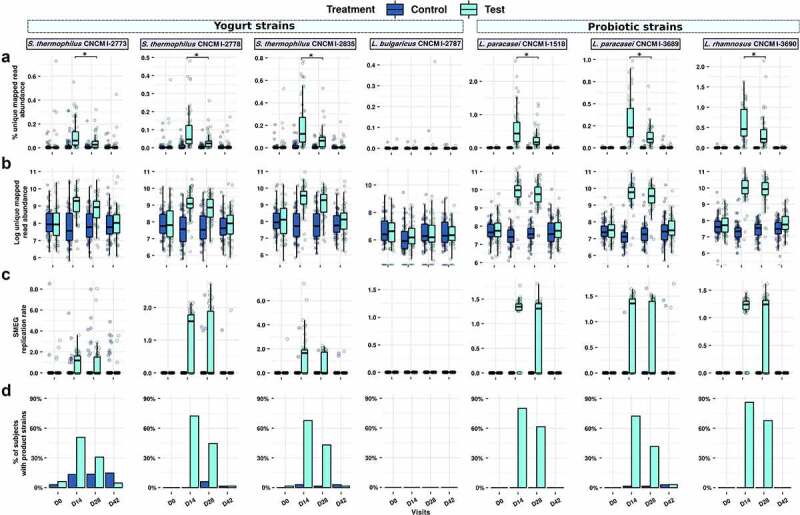
Strain-level detection and replication of product strains in the gut microbiota. a. Relative abundance of Test product strains were measured by mapping reads from each metagenome to the concatenated scaffolds of the seven product strains. Multi-mapped reads were excluded, and unique mapped reads were sum-scaled by the total reads in that sample to calculate percent abundance of product strains b. Percent unique mapped reads were further scaled by flow-cytometry based microbial cell counts for cytometry-scaled quantification of product strains. c. Rate of replication assessed by strain-level metagenomic estimation of replication rate (SMEG) scores d. Prevalence of strains detected based on replication rate. * denotes significant difference in test group between D14 and D28 timepoints (FDR < 0.05).

The test product is a fermented dairy drink containing three strains selected for their potential health effects, hereafter referred to as probiotic strains (*L. paracasei* CNCM I-1518, *L. paracasei* CNCM I-3689 and *L. rhamnosus* CNCM I-3690), and four yogurt strains (*L. delbrueckii* subsp. *bulgaricus (L. bulgaricus)* CNCM I-2787, *S. salivarius* subsp. *thermophilus (S. thermophilus)* CNCM I-2773, *S. thermophilus* CNCM I-2835, *S. thermophilus* CNCM I-2778). We used a strain-level metagenomic analysis for detection of the test product strains, where the shotgun reads from each sample were mapped to the whole genomes of the product strains in order to assess their relative abundance (Figure 2(a)). All strain abundances (except *L. bulgaricus* CNCM I-2787) increased during the consumption period from no detection at D0 to 0.05–0.47% (min-max) at D14, which in turn was higher than D28 (0.02–0.22%) (Wilcoxon rank-sum test, FDR < 0.001). Next, we scaled the mapped read abundance of the product strains by flow cytometry-based microbial cell counts to provide a quantitative abundance of the strains. These readouts were better correlated with previously conducted qPCR for *L. rhamnosus* and *L. paracasei* strains in these samples^24^ (Spearman’s rho: 0.58–0.66, p < .0001 versus Spearman’s rho: 0.28–0.36, p = .004–0.04 for unscaled abundances) (Figure S1). Similarly to unscaled (relative) abundances, cytometry-scaled (quantitative) abundance of product strains in fecal samples also showed that consumption of the fermented milk product led to a significantly higher quantitative abundance of all product strains (with the exception of *L. bulgaricus* CNCM I-2787) during consumption at D14 and D28 compared to baseline (Wilcoxon rank-sum test, FDR < 0.0001) (Figure 2(b)), but without no significant difference between D14 and D28 (Wilcoxon rank-sum test, FDR >0.05). At D42, 14 d after consumption ceased, all strains had returned to baseline level, with the exception of *L. rhamnosus* CNCM I-3690.

To complement the abundances of the product strains with their metabolic activity in the presence of antibiotics, we inferred their individual growth rates using a strain-level single-nucleotide polymorphism (SNP) based replication rate (SMEG) measurement. With this approach, an SMEG score of 1 indicates no replication rate, while a score of >1 indicates the fraction of the population making copy of their genome. For instance, a SMEG score of 1.25 indicates that 25% of the strain population is undergoing cell replication, and thus is metabolically active (Table S2). Of the seven product strains, only *L. bulgaricus* was below the detection limit of SMEG. The replication scores for the six observable strains increased significantly in the test samples compared to control samples, but only during the period of consumption (both at D14 and D28: Wilcoxon rank-sum test FDR <0.001 for all strains; D42: Wilcoxon rank-sum test not significant) (Figure 2c,d). The three probiotic strains *L. paracasei* CNCM I-1518, CNCM I-3689, and *L. rhamnosus* CNCM I-3690 were detected with replication scores of 1.23–1.35 (corresponding to 23–35% of cells engaged in active replication), in more than 73% and 63% of Test subjects at D14 and D28, with lower prevalence (42%) for *L. paracasei* CNCM I-3689 (Figure 2(c,d)). *Streptococcus thermophilus* strains are yogurt starters and are not generally thought to survive in the upper GI tract expected to be active. Surprisingly, the three *S. thermophilus* product strains had detectable growth rates in more than 52% and 31% of the Test subjects at D14 and D28, respectively, with median replication scores of 1.27–1.64 (27–64% of actively replicating cells), which mostly reduced to baseline levels at D42. In order to examine whether the enhanced replication rate for *Streptococcus* strains was specific to this study, we determined replication rates of *S. thermophilus* strains in fecal samples collected during a previous study with 40 healthy individuals who consumed the same fermented milk product but were not exposed to *Hp* treatment^25^ Considerably fewer subjects (up to 4/20 for CNCMI-2835 at D28) (Figure S2(a,b)) had detectable replication scores for *S. thermophilus* strains compared to those observed in the current study, suggesting that the *Hp* treatment increased replication rates of administrated *S. thermophilus*. We finally investigate whether the six strains that showed SMEG replication rate >1 (i.e. were actively replicating) were sensitive to antibiotics used during the study (amoxicillin and clarithromycin) either *in vitro* or by analysis of antibiotic resistance genes against 239 genes for macrolide and 3969 for beta-lactam. No resistance to antibiotics used in the study was detected based on criteria used for food bacteria by either method (Table S3).

### *Impact of* Hp *therapy and fermented product on microbiome composition and function*

**Figure 3.**
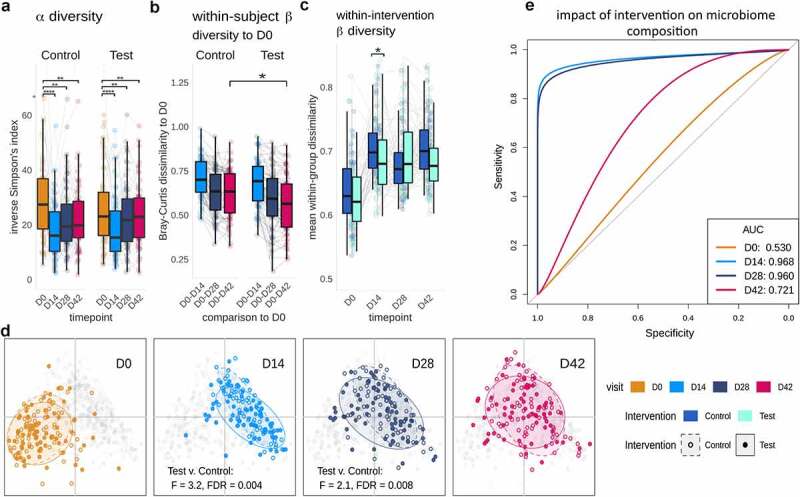
Species composition and diversity across trial. a. Inverse Simpson’s Index. Both groups showed significant changes in species-based alpha diversity (Wilcoxon rank-sum, FDR: 0.003 to <0.0001) throughout the trial, albeit with no significant differences between Test and Control groups. b. Within-subject beta diversity to baseline (D0) composition at D42 (Bray-Curtis dissimilarity, Wilcoxon rank-sum, FDR = 0.03). c. Within-group (between all Test, or between all Control subjects) beta diversity differences (Bray-Curtis dissimilarity, Wilcoxon rank-sum, FDR = 0,041) d. Principal Coordinates Analysis (PCoA) of Bray-Curtis dissimilarity shows a taxonomic shift immediately post-therapy (D0-D14), followed by significant differences between Test and Control composition at D14 and D28 (PERMANOVA, FDR = 0.004, 0.008 respectively), with a gradual but incomplete return toward a baseline-like state in both groups at D42 (overall D0-D42 difference in PERMANOVA, FDR = 0.001) E. Smoothed ROC curves for the classification of intervention (Control versus Test) at D0, D14, D28 and D42, showing very strong classification performance at D14 and D28, and a reduced degree of classification at D42.

Next, we evaluated the composition and functional response of gut microbiome. WMS analysis revealed a significant reduction in alpha-diversity (species level) through the *Hp* therapy followed by an incomplete recovery over the 4 weeks post-therapy (Figure 3(a) and Table S4). Differential abundance analysis revealed significant durable depletion in multiple taxa with respect to baseline, especially within the phyla Actinobacteria including *Collinsella aerofaciens* and multiple *Bifidobacterium* species (including *Bifidobacterium longum, Bifidobacterium adolescentis)* and Firmicutes (Figure S3).

A comparison of the Test and Control groups revealed that at D42, the fecal microbiota of Test subjects was more similar to their microbiota at baseline (D0; within-subject beta-diversity) (Figure 3(b)), Wilcoxon rank-sum, FDR = 0.033), indicating an accelerated recovery. Between-subject (within group) beta diversity was also significantly lower in Test group than in the Control group at D14 (Wilcoxon rank-sum, FDR = 0.041), suggesting that the fermented milk product induced less variability of gut microbiome composition between subjects than did the control product during *Hp* therapy (Figure 3(c)). In addition, beta-diversity analysis using Bray-Curtis dissimilarity showed a significant difference between the fecal microbiota in the Test and Control group during the intervention period (PERMANOVA at D14 and D28, FDR = 0.004, 0.008) (Figure 3(d), Figure S4, and Table S4).

A complementary method for distinguishing between groups of features involves machine learning using Extreme Gradient Boosting (XGB), which has proven efficacy for multi-dimensional microbiome data.^26^ As expected, XGB models could not distinguish between the test and control groups prior to consumption of the test product (D0), performing close to the mean for a binary classification (baseline AUC: 0.53). After consumption of the product, classification accuracy was high at 93.1% (AUC 0.96) and 92.1% (AUC 0.96) at D14 and D28, respectively (Figure 3(e), Figure S5). Interestingly, XGB could still distinguish between Test and Control groups with an accuracy of 66.96% and an AUC of 0.72 at D42 (Figure 3(e)), with features including for instance *Bacteroides cellulosilyticus* for Test group (Figure S5). Collectively, beta-diversity and machine learning analyses indicate that the consumption of the Test product promotes gut microbiota re-assembly after *Hp* therapy, with effects lasting beyond the time point when the product was consumed, and probiotic strains were detectable.

**Figure 4.**
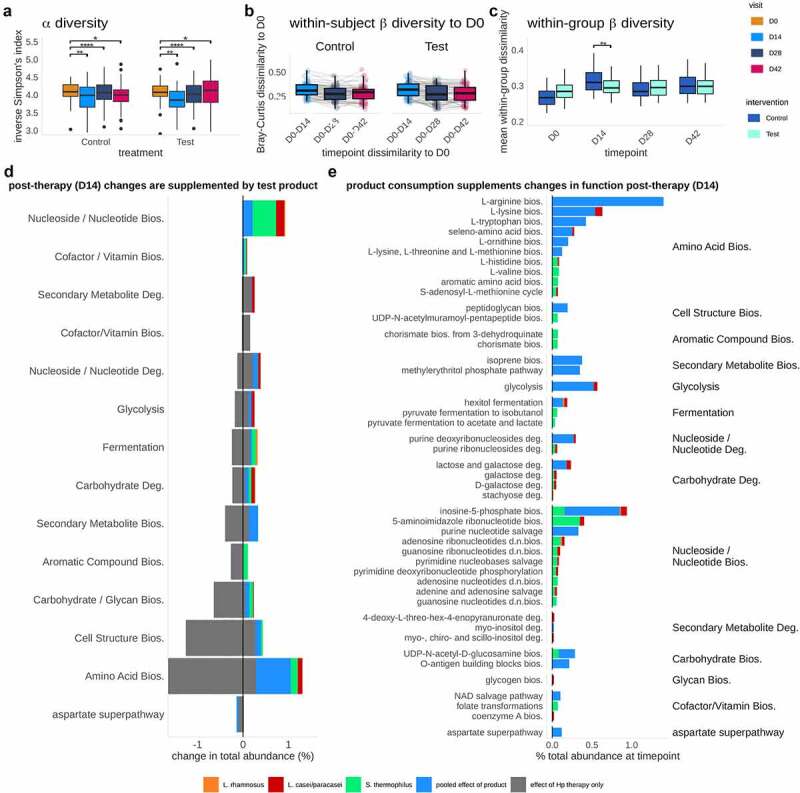
Product consumption alters functional diversity and composition, through both the addition of product strains and the enrichment of function in the total gut microbiome. a. Inverse Simpson’s Index of metabolic pathways. Alpha-diversity was reduced after *Hp* therapy and (D0 versus D28/D42; Wilcoxon test, FDR = 0.02). b. Within-subject beta-diversity to baseline shows a decrease in dissimilarity (i.e., increase in similarity) at timepoint D14-D42, however these trends were not significantly different between Test and Control (Bray-Curtis dissimilarity, Wilcoxon rank-sum, FDR = 0.8–1.0). c. Within-group beta-diversity dissimilarities at each timepoint (PERMANOVA), showing metabolic composition to differ significantly at D14 (FDR = 0.004). d. Grey: decreases (left) and increases (right) in total pathway relative abundances due to impact of *Hp* eradication therapy, at D14 with reference to D0 (FDR < 0.05); Colored bars: changes significantly associated with Test product consumption, contributed both directly from the product species (*L. paracasei*: red;*L. rhamnosus*: orange; *S. thermophilus*: green), as well as from the wider gut microbiome (‘pooled effect’ in blue; all FDR < 0.05). Apparent decreases in pooled pathway abundance represent stochastic differences in starting abundance (D0) between Test and Control, rather than decreases as a result of product consumption. e. Significant differences in total functional pathway abundance associated with Test product species at timepoints D14 (*L. paracasei*: red;*L. rhamnosus*: orange; *S. thermophilus*: green; pooled community functions: blue). Similar effects (panels D & E) are seen at D28 (Fig S6).

Having detected lasting effects on taxonomic composition from both the therapy and Test product, we were prompted to ask how these changes were reflected in metabolic functions of the fecal microbiota. Firstly, we assessed the same global diversity parameters as observed for taxonomic composition, using differential abundance analysis of MetaCyc metabolic pathways across the trial. This analysis showed that metabolic pathway alpha diversity (inverse Simpson’s index) decreased significantly post-Hp therapy (D0 versus D14; Wilcoxon test, FDR = 0.006) and remained below baseline levels at the end of trial (D0 versus D28/D42; Wilcoxon test, FDR <0.0001, FDR = 0.02) with no significant difference between intervention groups across the study (Figure 4(a) and Table S4). Although metabolic pathways in Test and Control did not differ in alpha-diversity or within-subject beta-diversity to baseline (Figure 4(a,b)), pathway beta-diversity within the Test group was significantly lower than in Control at D14 (Wilcoxon rank-sum, FDR = 0.02), indicating that product consumption led to a greater similarity in metabolic function in the Test group immediately post-therapy (Figure 4(c)). Similarly, we detected a dramatic change in the abundance of metabolic functions post-*Hp* eradication therapy, leading to both increases and decreases in a range of pathways involved in energy metabolism (biosynthesis of purines, cofactors, and electron carriers, fermentation, the citric acid cycle, and glycolysis), amino acid metabolism, and cell structure synthesis. Notably, these changes in functional abundance collate well with observed taxonomic decreases (e.g., depletion of *Bifidobacterium, Collinsella*, and *Clostridium*) and increases (e.g., establishment of *Eggerthella*) post-therapy (Table S6).

Following analysis of the overall function of the gut microbiome, we focused our attention specifically upon the pathways that were contributed by Test product species during therapy. To emphasize this comparison, we collated pathways that differed significantly at D14 (Figure 4(d)) and D28 (Figure S6) after product consumption in the Test group and pathways that differed significantly following *Hp* eradication therapy (Figure 4(d)) in the Control group. Pathways were filtered to include only those significantly affected by Test product consumption (FDR < 0.05), pathways within those same functional ‘classes’ (grouping categories as defined in MetaCyc’s hierarchy) as those affected by Test product consumption, and pathways that were significantly different to baseline at D14 or D28 in the Control group (CLR transformed values, FDR < 0.05; Figure S7). We found that product species contributed to multiple pathway classes that were depleted by *Hp* eradication therapy, e.g., carbon pathways (fermentation, glycolysis), biosynthesis (amino acids, nucleotides), and carbohydrate degradation (galactose, lactose, and stachyose) (Figure 4e). Test product consumption appeared to contribute a greater proportion of function at D14 than D28 (Figure S6), as observed with the relative abundance of the strains, which is likely the result of the recovery of the background microbiota. Some increases in pathway abundance were species-specific, e.g., folate production by *S. thermophilus*, stachyose degradation by *L. rhamnosus*, and L-lysine biosynthesis by *L. paracasei*. In addition to supplementation through these species-specific pathways, consumption of the Test product induced larger changes in ‘pooled’ or community-level pathway abundances (i.e., not directly attributable to any single taxon or of ambiguous classification), indicating that product consumption elicits a broader response in gut microbiome function. Overall, this analysis revealed a measurable enrichment of functional pathways due to Test product consumption that are depleted by *Hp* therapy, suggesting that the introduced species contributed transiently to various metabolic pathways, although the effects of which were limited to consumption period.

### Product strains are associated with alpha diversity-based recovery in the gut microbiome

**Figure 5.**
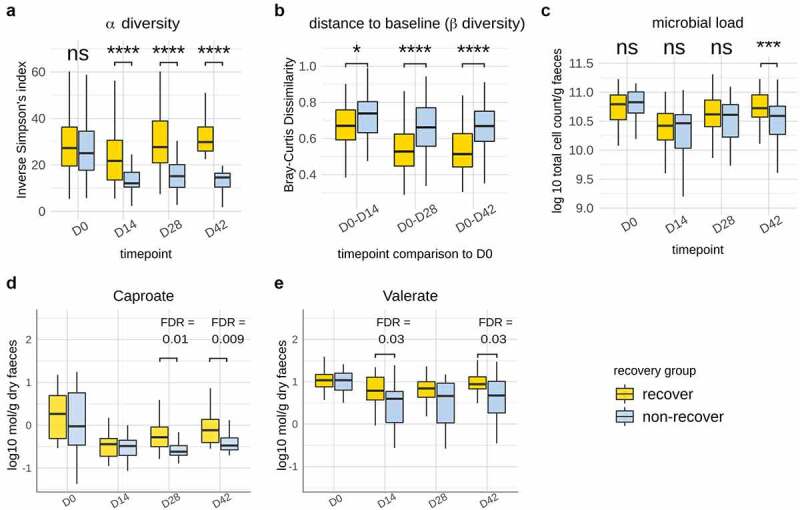
Identification and characterization of gut microbiota recovery following Hp eradication therapy. a. Dynamics of alpha-diversity in participants stratified into “recovery” and “non-recovery” groups based on ‘median boundary’ species-level Inverse Simpson Index at D42. b. Within-subject dissimilarity to baseline (Bray-Curtis) was significantly lower in the “recovery” group throughout the duration of the trial c. Microbial load (Log 10 cell count/g fecal samples, flow cytometry) was significantly lower in the non-recovery group. Amounts of caproate (d) and (e) valerate (Log10 mmol/g dry fecal weight) were more abundant in Recovery subjects at D28 & D42 and D14 & D42, respectively. Abbreviations: FDR: false discovery rate; **: FDR < 0.01; ****: FDR <0.0001.

Given that the fermented milk product had a measurable impact on compositional recovery of the microbiota post *Hp* treatment, we were prompted to investigate the contribution of individual strains. To achieve this, we stratified subjects using alpha diversity at D42 (species-level Inverse Simpson’s index) as employed previously.^27^ Recovery of the microbiome post-treatment was defined in reference to a median ‘boundary’ (defined as the median Inverse Simpson’s index ± 10% interquartile range) at D42. Out of the 135 subjects, 62 presented an Inverse Simpson’s index above the median boundary, 57 presented an alpha diversity below the boundary, while 16 subjects fell within the boundary and were not considered for this analysis. This definition allowed clear separation of two groups in terms of recovery, illustrated by subjects below the boundary remaining significantly below their baseline alpha diversity at D42 (Wilcoxon signed rank test, FDR < 0.0001), while those above the boundary were, on average, slightly above their baseline value. Subjects above the partition were therefore designated as “recovered” in terms of alpha diversity post-therapy, whereas subjects below assigned a “non-recovered” state during the timeframe of the study. Inverse Simpson’s Index did not differ between these groups at baseline but was significantly lower from D14 onwards in the non-recovery group (Wilcoxon rank-sum test, FDR < 0.0001) (Figure 5(a)). Further comparison of within-subject beta-diversity (shift from baseline) across time points confirmed that gut microbiota from subjects in the recovery group were closer to baseline (D0) composition at all time points post-therapy (Wilcoxon rank-sum test, FDR from 0.028 to <0.0001) (Figure 5(b)). Additionally, the recovery group was characterized by a significantly higher microbial load in samples at D42 (Wilcoxon rank-sum test, p = 0.0002) as determined by flow cytometry (Figure 5(c)). We previously showed in a subset of subjects (N = 73) the partial recovery after *Hp* treatment of SCFA (acetate, propionate, butyrate, valerate, and caproate), BCFA (iso-valerate, iso-butyrate), and the recovery, although variable, of calprotectin, a host marker of inflammation.^24^ Here, we investigated whether their level differed based on gut microbiome recovery status. Of these, valerate (D14, D42) and caproate (D28, D42) were more abundant in recovery (FDR = 0.009–0.03) (Figure 5(d,e)), while other parameters did not differ discernibly (Figure S8). As expected, the abundance of multiple species differed between recovery and non-recovery groups at D42 compared to baseline (Table S7). Among them, *Faecalicatena gnavus*, the spore former *Clostridium bolteae*, and several species from *Blautia* were more abundant in non-recovery, which were also found to be durably enriched following antibiotic intake in previous studies.^4,10^

**Figure 6.**
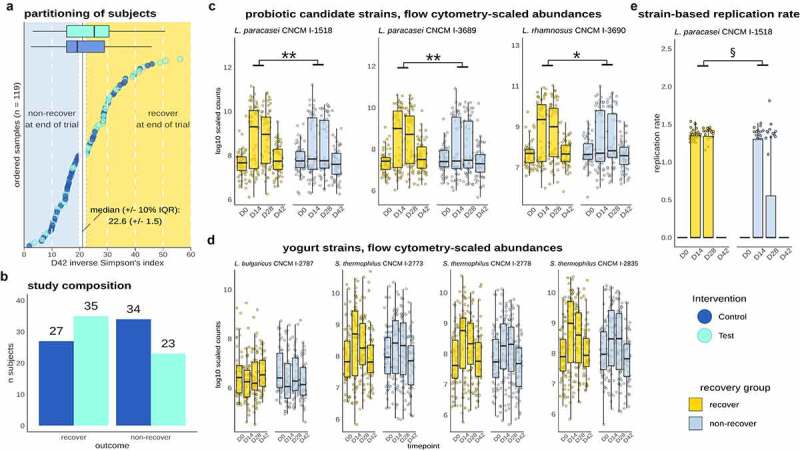
Recovery was linked to consumption of the Test product, abundance of probiotic strains, and *in vivo* replication of *L. paracasei* CNCM I-1518. a. Partitioning of recovery/non-recovery based on D42 alpha diversity shows unevenly distributed subjects across Test and Control groups, with a higher number of recovered subjects in the Test group although not significant (p = 0.1). b. Number of subjects from Test and Control in the recovery/non-recovery groups. c. Flow cytometry scaled abundances of Test product strains. *L. paracasei and L. rhamnosus* strains abundance was higher in recovery compared non-recovery groups. d. Flow cytometry scaled abundances of yogurt strains e. Replication rates of *L. paracasei* CNCM I-1518 was higher in the recovery (*: FDR < 0.05;**: FDR < 0.01; ****: FDR < 0.0001; p < 0.05: §).

We subsequently asked whether recovery was linked to consumption of the Test product and the establishment and *in vivo* activity of individual strains. There was a trend for a higher proportion of Test subjects in the recovery group (N = 35, 60%) than in the non-recovery group (N = 23, 40%) (Chi2 test, p = 0.1) (Figure 6(a,b)). Cytometry-scaled strain abundances for *L. paracasei* CNCM-1518, *L. paracasei* CNCM-3689, and *L. rhamnosus* CNCM-3690, but not the yogurt starter strains (*L. bulgaricus, S. thermophilus*), were significantly higher in the recovery group (FDR <0.05) (Figure 6(c,d)). Finally, we explored whether the replication rate of strains was differentially associated with gut microbiota recovery. Out of the six strains that were shown to replicate, *L. paracasei* CNCM-1518 replication rate was significantly higher in the recovery group (p value = 0.03) (Figure 6(e)). Collectively, these findings suggest that recovery of gut microbiome diversity is linked to an increased quantitative abundance of *L. paracasei* CNCM-1518, *L. paracasei* CNCM-3689, and *L. rhamnosus* CNCM-3690 strains, and a higher metabolic activity of *L. paracasei* CNCM-1518.

## Discussion

Probiotics are efficacious in the treatment of antibiotic-associated diarrhea (reviewed in^28^), but it is unclear how they aid the restoration of the microbiome after antibiotics. We recently showed that the consumption of a multi-strain product consisting of yogurt and probiotic strains induces a faster recovery in subjects that undertook *Hp* eradication therapy, which was reflected by lower within-subject beta-diversity dissimilarity to baseline, enhanced short-chain fatty acids, and compositional differences in the fecal microbiota post-*Hp* treatment.^24^ Here, we used metagenomic approaches with strain-level resolution to determine the contribution of the individual bacterial strains to structural and functional recovery.

As of yet, large-scale studies focusing on metagenomic-based growth rate estimation often examine the population-level behavior at species level,^27,29,30^ and strain-specific growth rates from metagenomic datasets are understudied.^16,31^ In our study, SMEG allowed us to identify strain-specific unique SNP patterns in the core genomic regions of several, very closely related strains, providing new insights into the strain-level interactions.^16^ All strains, except *L. bulgaricus*, were found to be increased transiently in terms of abundance and replication levels. Strains of *S. thermophilus* are used as starter cultures in classic yogurt fermentations and are commonly detected in fecal samples from yogurt consumers,^32^ but show poor survival for upper GI tract conditions.^33^ Interestingly, in an alternate study that tested the same milk product without *Hp* therapy in healthy people, the replication rates of the same *S. thermophilus* strains were much lower in the gut microbiome although the daily doses of the strains administered were higher.^25^ This suggests that *Hp* therapy enhances the ability of yogurt starters to survive in the gut, possibly directly through a lowering of gastric pH through the PPI therapy^34,35^ or indirectly by lowering colonization resistance induced by the antibiotic treatment. We found that strains of the fermented milk product did not exhibit resistance to the antibiotics tested. The recovery of Test product strains in the feces despite their sensitivity to the antibiotics used might be explained by a combination of a rapid intestinal absorption of antibiotics and a continuous product consumption (twice daily), resulting in a detectable amount of living strains in the gut. While the probiotic strains were detected only during the consumption period, a significant lasting effect of the Test product on gut microbiota assembly was detected during the follow-up phase using a machine learning approach (XGB). To our knowledge, this is the first time that such an approach has been conducted to assess the effect of a probiotic product on gut microbiota assembly during recovery from a perturbation.

Next, we wondered whether recuperation of the gut microbiota post-therapy was reflected by changes in function, and whether a role for Test product species was apparent. Here, a more limited effect was observed in response to consumption of the Test product, restricted to the intervention period. During the consumption of the product, functions that differed between groups were contributed by both Test product species *S. thermophilus, L. paracasei*, and *L. rhamnosus* and others. Notably, among functions that were altered following therapy, some were contributed by *S. thermophilus, L. paracasei*, and *L. rhamnosus*, largely in amino-acid synthesis metabolic pathways, representing the pathway class most significantly altered following therapy. *S. thermophilus* contributed to pathway related to folic acid synthesis, which cross-feeding, was shown to stimulate butyrate producers,^36^ and potentially other members. Similarly, *L. paracasei* contributed to gut microbiota function through synthesis of some amino-acids (including lysine, histidine, and methionine), which may be used for butyrate production.^37^ As the Test product had a higher effect on gut microbiota recovery at a compositional level compared to functional level, we subsequently studied inter-individual variability in recovery by stratifying participants based on composition-based alpha-diversity at D42 as compared to baseline. This analysis revealed that valerate and caproate were increased in the recovery group. Valerate, which was increased through the test product,^24^ has been previously associated with high microbial richness.^38^ Valerate is an end product of amino acid fermentation, and its implications for gut health are poorly studied. Interestingly, a previous study showed the capacity of valerate to inhibit *C. difficile* in an *in vitro* gut model of the microbiome following antibiotic exposure.^39^ However, its effect on gut microbiota recovery was not tested, and future studies are warranted to study its role, as well as other potentially involved metabolites such as bile acids^40,41^ .

Stratification of subjects revealed that microbiome alpha-diversity-based recovery was also linked to the *in vivo* abundance and activity of probiotic strains. The three probiotic strains had a higher abundance in subjects that were stratified into the recovery group. Although cause and effect cannot be assigned to these associations, ecological considerations suggest a functional role of the probiotic strains in recovery. The recovery group showed both a significantly higher alpha diversity and bacterial load, which is likely linked to an increased metabolic activity and higher colonization resistance. Although this would result in a higher competitive pressure for the incoming strains, the abundance of the probiotic strains was elevated. In addition, only the probiotics, but not the yogurt strains, were elevated in recovery. We therefore conclude that the higher abundance of the probiotic strains is unlikely to be the indirect result of the enhanced microbiome recovery but instead points to a direct contribution of these strains. Interestingly, *L. paracasei* CNCM I-1518 was the only strain in which replication rate was associated with gut microbiome recovery, possibly due to a higher fitness of this strain in the gut. This strain, in a fermented milk matrix, has been well studied in the context of infection at both systemic and local (gastrointestinal) sites (as reviewed in^20^), but little for its effect on gut microbiota either in humans^42,43^ or *in vitro*.^36,44,45^ Future studies are warranted to identify precise mechanisms of the ecological contribution of three probiotic candidates and specifically *L. paracasei* CNCM I-1518 to gut microbiome recovery using complementary approaches such as genome-scale modeling, which allow to identify inter-strain/species metabolic interactions in the gut microbiome.

## Conclusion

By using a high-resolution metagenomic approach, we have shown that probiotic strains administered with a fermented food product are linked to a significant acceleration in recovery from *Hp* eradication therapy. Recovery of alpha-diversity was linked to differences in specific metabolites, specifically valerate, that these probiotic strains could contribute to. To our knowledge, this is the first study that links improvements in microbiome recovery, although small, after antibiotics by probiotics to the abundance and *in vivo* activity of *L. paracasei* CNCM I-1518, thereby providing preliminary evidence for their contribution to the effects. In future studies, an even broader analysis of gut microbiota recovery following antibiotics, considering various ecological markers is warranted. Taken together, this study expands our understanding of the ecological role of lactic acid bacteria on the gut microbiota following antibiotic intake, and especially the variable contribution of probiotic strains toward recovery. While the effect we observed is rather small, our study opens opportunities for 1) specifically gut microbiota-powered clinical trial, 2) identification of ingredients to increase the colonization of tested strains (synbiotics), and 3) rationally driven identification of other strains, specifically those with more durable alteration such as *Bifidobacterium* species, to alleviate the collateral damage related to antibiotic treatment.

## Material and methods

### Clinical study

The study was monocentric, randomized, double blind, and controlled, with two parallel arms (Test/Control, allocation ratio: 1–1). As described in Figure 1, the study included a screening phase, 14 d of *Hp* eradication treatment (D0-D14), 28 d of product consumption (D0-D28), and 14 d of follow-up (D28-D42), with dietary restriction (D0-D42) (no yogurts, probiotics in fermented dairy products or supplements). Seven visits were planned, in a clinical unit (Charité Research Organization GmbH, Berlin, Germany): for inclusion (V1), randomization (V2-D0), and evaluation (V3-D7 to V7-D42), with stool sampling at D0, D14, D28, and D42. Subject screening lasted from September 16, 2016 (first inclusion [Informed Consent Form signature]) to May 31, 2017 (Last inclusion), and the study experimental phase from October 7, 2016 (first randomization) to August 10, 2017 (last visit). The main inclusion criteria were as follows: *Hp* infection, based on positive^13^C-Urea Breath test^46^ and at least one positive Urease or *Hp*-gastritis histological test; dyspepsia with medical prescription for a *Hp* eradication triple therapy; age 18–65 y and a body mass index (BMI) of 19 to 30 kg/m^2^. Test product was a fermented milk containing probiotic strains *L. paracasei* CNCM I-1518 (formerly known as DN 114–001), *L. paracasei* CNCM I-3689, and *L. rhamnosus* CNCM I-3690 strains and four yogurt strains (*L. bulgaricus* CNCM I-2787, *S. thermophilus* CNCM I-2773, *S. thermophilus* CNCM I-2835, *S. thermophilus* CNCM I-2778). Control product was an acidified non-fermented milk product, depleted in lactose, containing phosphoric acid and carboxy methyl cellulose. Both products were manufactured by Danone Research, France. Subjects ingested one bottle twice daily (100 g/bottle) of Test or Control product per day for 28 d (one at breakfast, one at dinner). For *Hp* eradication, subjects were treated by a triple therapy (ZacPac®, Takeda, Singen, Germany) including a PPI (pantoprazole 40 mg), and two antibiotics (clarithromycin 500 mg and amoxicillin 1000 mg), twice daily, for 14 d.

### *Antimicrobial susceptibility testing and* in silico *prediction of antimicrobial resistance genes*

Minimum inhibitory concentrations (MICs) of amoxicillin and clarithromycin were determined by broth microdilution method in accordance with the standard ISO 10932 (2010) using pre-coated microtiter plates. Microbiological cutoff values were adopted by EFSA and for those antimicrobials not covered by EFSA, from EUCAST. ARGs were predicted using AMRFinderPlus (version 3.10.1, with Database version: 2021–03-01.1)^47^ against 239 genes for macrolide and 3969 for beta-lactam. The presence of ARG was performed based on EFSA recommendation for microorganisms intentionally used in the food chain,^48^ i.e., resistance when the % Coverage is >70% and % Identity is >80%.

### Fecal sample collection and shotgun sequencing

Fecal samples were collected in the study from 135 (67 in Test and 68 in Control group) randomized subjects at four time points (D0, D14, D28, and D42).^24^ Total cell count (g/wet weight) was assessed using flow cytometry as previously described.^24^ DNA was extracted based on standardized protocol Q,^49^ a protocol that has been shown to better retrieve gram-positive bacteria,^50^ and quantified by fluorimetry with the Qubit dsDNA HS Assay Kit (Thermo Fisher Scientific). Sequencing libraries were prepared with the Nextera XT DNA sample preparation kit, according to the manufacturer’s instructions. Library Profile and concentration were evaluated with the Agilent High Sensitivity DNA Kit in an Agilent 2100 Bioanalyzer. Libraries were pooled in sets of 14 to 16 samples, and each pool was then run on a NextSeq500 sequencer (Illumina), in the 150 bp paired-end read configuration, with the NextSeq® 500/550 High Output Kit v2, in accordance with the manufacturer’s instructions (NextSeq System Suite v2.1.2 2017). The PhiX Control library (v3) (Illumina) was combined with the amplicon library (expected at 1%).

### Bioinformatic analysis

Initial quality control (QC) of sequences was carried out using the packages FastQC and MultiQC to generate and summarize sequence quality output prior to quality filtering and processing. Sequences were processed in Trimmomatic (version 0.39) to remove low quality reads and read-portions, as well as excising any remaining scaffold sequences from the Illumina sequencing platform (argument ILLUMINACLIP). After initial QC steps, files were reevaluated using FastQC and MultiQC to ensure that any issues had been resolved. Raw FASTQ output from sequencing averaged 64 million reads (±22%) per sample. Removal of host and contaminant sequences was carried out using Kneaddata (version 0.7.2) to identify sequences from the human genome (human genome build hg37) and known human contaminant elements.

After trimming or fully removing DNA sequences (“reads”) below a quality threshold of Q = 26, and purging of sequences considered “contaminant,” samples were reduced by approximately 6% to an average of 61 million reads (±27%), giving a total of 33.5 billion reads across all samples. Kraken 2 (version 2.0.8) assigned taxonomy to reads through comparison of sequence k-mer frequencies using the Ecogenomics GT Database (release 89); the abundances of identified taxa were then estimated using Bracken.^51^ Separately, the HUMAnN2^52^ (version 2.8.1 pipeline) allows characterization of aligned reads to pre-computed reference databases (full chocophlan database plus viral sequences, v0.1.1; uniref90 database v. 1.1), and then compiled the tallied gene abundances to determine whether pathways were fully represented within samples (coverage of MetaCyc metabolic pathways and superpathways),^53^ what microbes contributed those pathways (pathway source organism), and at what frequencies those pathways were present (MetaCyc metabolic pathway abundance). The set of pathway features was subset to include only those with an assigned function for further analysis.

### Strain tracking analysis using detection and estimation of growth rate

We used FastANI to calculate Average Nucleotide Identity (ANI) between whole genomes of product strains.^54^ Three *S. thermophilus* product strains and two *L. paracasei* had an ANI of >98% and 98.15%, respectively. Moreover, ANI comparison between three *S. thermophilus* product genomes and three complete S. *salivarius* genomes (GCF_000785515.1, GCF_002094975.1, GCF_016127535.1) revealed 89–90% sequence similarity. In order to determine relative abundance of the consumed probiotic strains, reads from each metagenome were mapped to the scaffolds of seven product strains (*L. paracasei* CNCM I-1518, *L. paracasei* CNCM I-3689, *L. rhamnosus* CNCM I-3690, *L. bulgaricus* CNCM I-2787, *S. thermophilus* CNCM I-2773, *S. thermophilus* CNCM I-2835, and *S. thermophilus* CNCM I-2778) using bbmap v38.92 with 100% sequence identity and perfect mode enabled. Multi-mapped reads were excluded and only reads mapping uniquely to the scaffold of product strains were considered. This approach ensures that reads mapping to conserved portion of the product strains are excluded and only reads mapping to strain-specific portion of the product strains are considered. Percent abundance of product strain was further scaled by flow cytometry-based microbial cell count.

Strain-level metagenomic estimation of growth rate (SMEG) was used for inferring replication rates and colonization ability of the seven product strains. The SMEG pipeline uses a differential coverage pattern of SNPs across the origin of replication (*ori*) and terminus (*ter*) sites of the reference genomes, to estimate the growth rates of closely related bacterial strains. In addition, SMEG uses differential coverage patterns of SNPs identified within the universally conserved “core” genes of closely related reference strains, to increase strain-specificity. A database of strain-specific differentiating SNPs for each species of interest (i.e., product strains) was created, followed by a *de novo*-based approach to estimate the growth rate in Test and Control samples (Table S8). Briefly, 32 genomes of *Streptococcus thermophilus* were retrieved and used to construct a species-specific database using an SNP assignment threshold of 0.5 with iterative clustering, identifying 8 clusters. Each of the three product strains belonging to *S. thermophilus* were assigned to three respective clusters (CNCM I-2773: Cluster-4, CNCM I-2778: Cluster-5, CNCM I-2835: Cluster-6); strains were considered present or actively replicating in each sample based on the detection of the corresponding cluster. Similarly, we downloaded 35 strains of *L. paracasei* from NCBI, and constructed a species-specific database using an SNP assignment threshold of 0.8 with iterative clustering and detected 8 different clusters. The two different product strains from species *L. paracasei* CNCM I-1518 & CNCM I-3689 were assigned to Cluster-1 (CNCM I-1518) and Cluster-3 (CNCM I-3689), respectively. Next, we downloaded 63 strains of *L. rhamnosus* and 21 strains of *L. bulgaricus* from NCBI and constructed species databases using iterative clustering and an SNP threshold of 0.9 for *L. rhamnosus*, and 0.6 for *L. bulgaricus*. SMEG identified 10 and 7 different clusters for *L. rhamnosus* and *L. bulgaricus*, respectively. The product strains from the species *L. rhamnosus* were assigned to Cluster-1 (CNCM I-3690), while *L. bulgaricus* product strain was assigned to Cluster-6 (CNCM I-2787). Test and control samples were analyzed for detection of product strain-specific clusters using *the de novo*-based approach with a default parameter of cluster detection threshold (-d) of 0.2 and a sample-specific SNP assignment threshold (-t) of 0.6. For improved accuracy of growth rate measurements, we only reported clusters with non-zero SNP sites (-u) of ≥100 and SNP coverage (-c) threshold of ≥5X.

As a validation of our approach, we also estimated the replication rate of the three *S. thermophilus* product strains in metagenomes from a cohort of 40 healthy subjects, who were not exposed to Hp treatment or antibiotics and who consumed control or the test product for a period of 1 month.^25^ Briefly, raw fastq files were downloaded using the fastq-dump script in SRA Toolkit and were processed and analyzed as described above. We processed 103 valid SRA runs associated with the NCBI Bio project No PRJEB35769 that consisted of an average of 43.2 ± 8.65 million quality filtered reads. These metagenomes were further analyzed for detection of *S. thermophilus* strain-specific clusters as described above. To evaluate the difference in product strain detection and rates of strain replication between intervention groups, scaled unique mapped reads abundance and SMEG values were compared between Test and Control using a Wilcoxon rank-sum test at timepoints D14, D28, and D42, with correction for multiple testing using the Benjamini–Hochberg method.

### Dosage of short-chain/branched-chain fatty acids and calprotectin

Short chain fatty acids (SCFA: acetate, propionate, butyrate, caproate, and valerate) and branched-chain fatty acids (BCFA: isobutyrate, isolavelarate) were analyzed in the last 61 subjects randomized. SCFA and BCFA concentration in stools (per gram of dry feces) was measured by headspace gas chromatography mass spectrometry (GC-MS) (column Rx-624Sil MS, RESTEK, France). Calprotectin was analyzed in the last 73 subjects randomized. Calprotectin concentration in feces was measured by ELISA (PhiCal Calprotectin ELISA, Immungiagnostik AG, Germany).

### Statistical analysis

All diversity and differential abundance analyses were performed using R version 3.6.

#### Alpha and beta-diversity

For taxonomic, and metabolic pathway features, alpha diversity metrics (Shannon’s H, Inverse Simpson’s Index, and feature richness) were calculated via the R package vegan (function *richness*). As residual errors remained non-normally distributed (Shapiro–Wilk test) after Box-Cox transformations (function car::*bcPower*) and removal of outliers based on Cook’s distance (function *cooksd*), alpha diversity indices were tested using the non-parametric Wilcoxon signed rank (for paired data) and rank-sum (for unpaired data) tests. For each alpha diversity metric, differences were assessed between groups by timepoint and between all pairwise-combinations of timepoint irrespective of group membership. For each comparison, p-values were corrected using the Benjamini–Hochberg method over all tested features.

For beta diversity of taxonomic and metabolic pathway features, homogeneity of variance was evaluated using function *betadisper* (package vegan), while permutational testing of variance (PERMANOVA) was performed using function adonis2 (package vegan) at each timepoint between groups and separately between timepoints irrespective of group. A within-subject analysis was performed comparing dissimilarity to baseline for D14, D28, and D42 between the two groups using the Wilcoxon rank-sum test. Within-group dissimilarities were compared using the same test, based on each participants’ mean dissimilarity to fellow group members, at each timepoint, respectively. For each metric, *p*-values were corrected within each set of comparisons using the Benjamini–Hochberg correction at an FDR cutoff of p < .05. Comparison of beta diversity composition was performed using a Mantel test of Spearman rank-correlation between taxonomic and pathway Bray-Curtis dissimilarities, with a significance estimated over 1001 permutations.

#### Differential abundance testing

Features were centered log-ratio (CLR) transformed with count-multiplicative replacement of zeroes as separate feature tables (microbial abundance, pathway abundance) before prevalence filtering at an abundance of 0.0001 (0.01%) in 0.1 (10%) of samples in the raw feature table. Feature sets were then treated as a single dataset for differential abundance testing using linear mixed-effects models (package lme4) of the form *feature ~ visit + group + random effect (subject)*, invoking appropriate contrasts for post-hoc tests (function ANOVA). Within each set of comparisons, multiple testing correction was performed using the Benjamini–Hochberg control for the false discovery rate (FDR, function p.adjust), with the alpha risk set at 0.05. For graphical representations and where noted, the difference in CLR abundance of feature abundances were calculated from the timepoints under consideration in that comparison. The package Complexheatmap^55^ was used to create community heatmaps of double z-scored, CLR-transformed features using the 30 most relatively abundant taxa (rows clustered on 1 – Spearman correlation between taxa, with Ward’s D2 hierarchical clustering via hclust (package stats)) in Control samples (columns clustered using Ward’s D2 of Bray-Curtis dissimilarities via hclust (package stats)).

### Definition of recovery and non-recovery groups

Subjects were stratified based on post-Hp treatment alpha diversity based on the median value of the Inverse Simpson’s Index at D42 for species features (20.5 in this study), with an exclusionary margin of 10% of the inter-quartile range (i.e., an exclusionary margin of ±1.64 about 20.5) above and below the median.^27^ In addition, final (D42) Inverse Simpson’s index values were compared to the respective baseline values (D0) to determine whether alpha diversity was significantly different between the start and end of the trial for either group (Wilcoxon signed-rank test) (and further reported as recovery and non-recovery). Tests were corrected for multiple testing correction (Benjamini–Hochberg method). Differences in the partitioning of intervention groups (Test, Control) to recovery groups (recover, non-recover) were tested using a Chi^2^ test with p values generated through permutations (n = 2000). Alpha and beta diversity of Recovery/Non-Recovery groups were compared using the same methods and parameters previously described in this study, while recovery/non-recovery differences were tested for various variables (BCFA, SCFA, calprotectin, strains, and rate of replication) were tested through Wilcoxon rank sum test as reported above and the Dunn test when comparing between more than two groups.

### Machine learning

Gradient-boosted tree models for the classification of Intervention groups at individual timepoints were generated with xgboost [1.4.1.1 (https://CRAN.R-project.org/package=xgboost)] using taxonomic features (filtered to those present in at least 10% of samples, with no minimum abundance). Both classification and prediction were performed with a nested loop approach: The inner fivefold loop was used for feature selection (mean CLR difference between groups of >1), with determination of optimal model parameters via random grid search with 100 iterations (Group classification); the outer leave-one-out loop was used to predict or classify the state of the *nth* sample. Model performance was evaluated based on classification or prediction accuracy and the area under the receiver operating characteristic (AUROC) via the pROC package [1.17.0.1,^56^]. Feature importance was calculated as the average gain when added to a model, multiplied by the frequency of inclusion in a model.

## Supplementary Material

Supplemental Material

## Data Availability

The sequence data for the project are publicly available through the European Nucleotide Archive (https://www.ebi.ac.uk/ena/) under accession number PRJEB52093.

## D eclaration of interest

E.G., F.S., P.V., and M.D. are Danone Nutricia Research employees. M.D. is inventor of patent applications (WO2015159125A1, WO2015159124A1) dealing with the use of *L. paracasei* and *L. rhamnosus* in the context of antibiotic. J.W. is a co-owner of Synbiotic Health, a developer of synbiotic products. M.J.C. is co-founder and Chief Executive Officer of SeqBiome Ltd. and scientific consultant for Second Genome and Mars PetCare. M.J.C. has received a research grant from Danone Nutricia Research. All other authors have no competing interests.
