# Supplementary material for: Improved gut microbiome recovery following drug therapy is linked to abundance and replication of probiotic strains
Source: Gut Microbes. 2022 Aug 2;14(1):2094664. doi: 10.1080/19490976.2022.2094664 (PMC9348039; doi:10.1080/19490976.2022.2094664)
Supplement: Supplemental Material [file KGMI_A_2094664_SM2715.zip › FitzGerald_2022_Supplementary material_VF.docx]

**
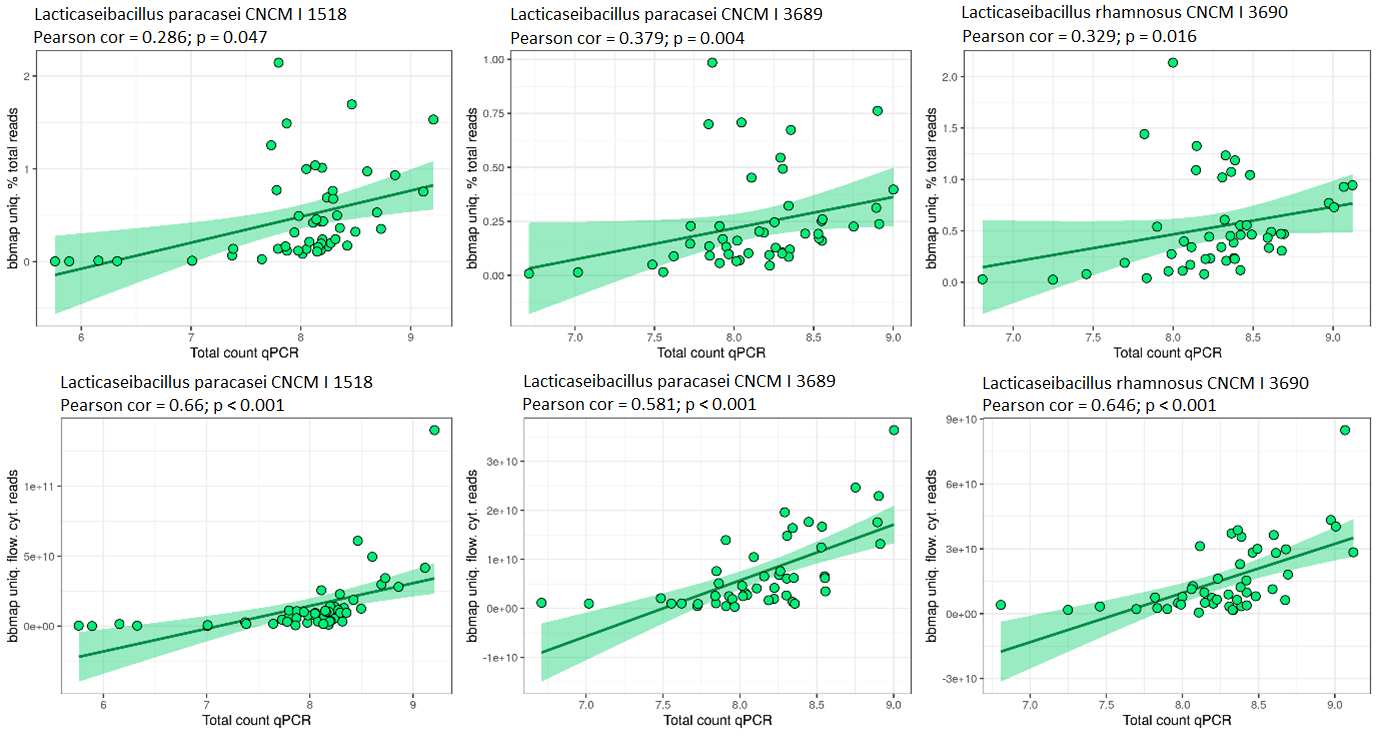
**

**Figure S1. Correlations analysis between strain-specific total qPCR counts and percent/ flow-cytometry scaled unique mapped read abundance**


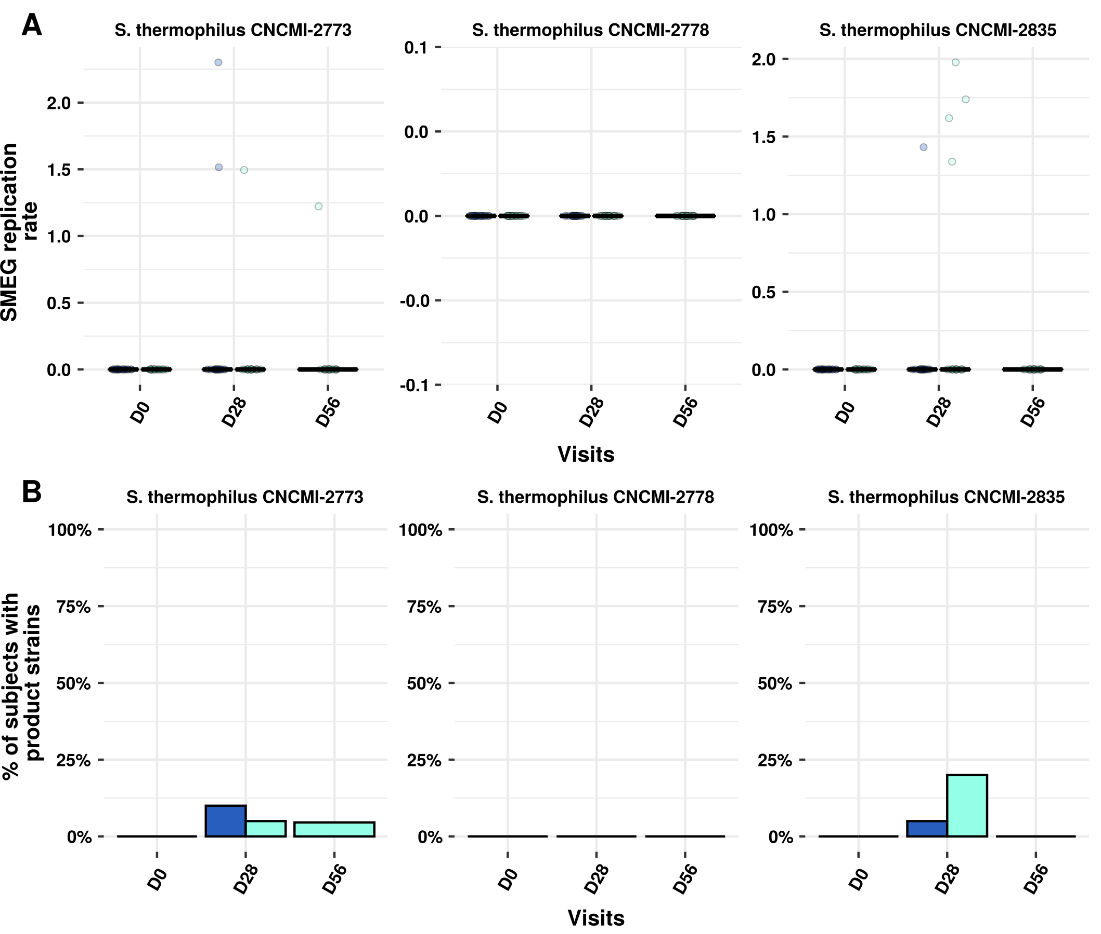


**Figure S2. Replication rates of *S. thermophilus* product strains in the gut microbiota from healthy subjects from a previous study. A.** Rate of replication assessed by SMEG score and **B.** prevalence of the three *S. thermophilus* strains detected based on replication rate in healthy subjects who were not exposed to Hp challenge^25^ (NCBI Bio project PRJEB35769). CP.3 denotes Control and Test.3 denotes Test participants


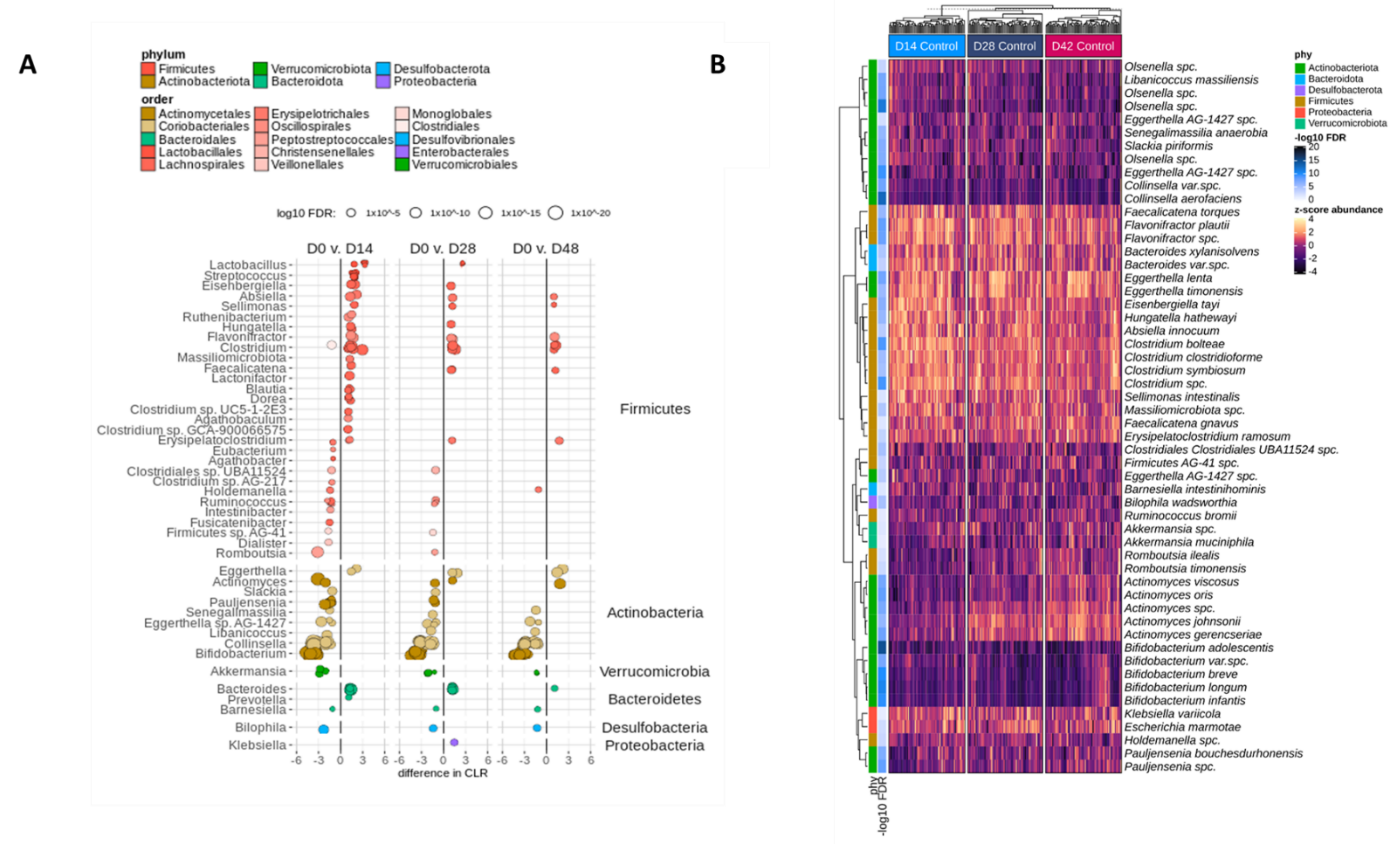


**Figure S3.** **Effect of Hp therapy on gut microbiota composition in control group. A.** Dot plot of species differentially abundant at D0:D14, D0:D28, or D0:D42 in Control, arranged at genus level for legibility. X axis reflects modelled difference in abundance (CLR) from baseline, while size of point reflects significance (-log10 FDR). Positive value in CLR indicates higher prevalence at the given time point (D14, D28, D42) versus baseline **B.** Heatmap of the most differentially abundant species at D0:D14, D0:D28, or D0:D42, showing change in CLR abundance with respect to baseline (CLR difference). The most significant differences from baseline are persistent reductions in the number of Actinobacteria (*Bifidobacterium, Collinsella*), and increases in *Eggerthella*, *Bacteroides*, and several *Firmicutes* taxa. Higher values for CLR abundance (trending towards orange) indicate a higher log-ratio to the mean abundance of that sample, while double z-score standardisation brings values within comparable ranges of values, both within samples and within features. Row annotations: Spearman rank-correlation clustering (Ward’s D2), feature phylum, and –log_10_ FDR value of differential abundance. Column annotation: sampling timepoint, and Ward's D2 hierarchical clustering, based on Bray-Curtis distance


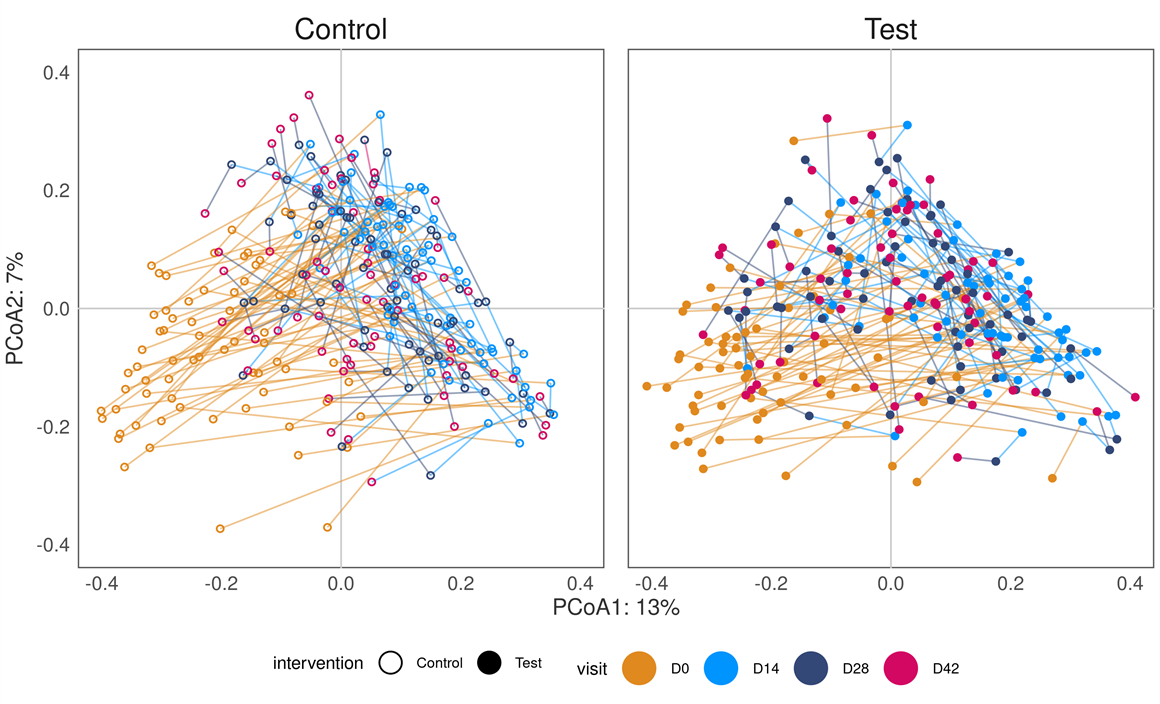


**Figure S4.** Principal Coordinates Analysis (PCoA) of changes in subject-level microbiome composition during course of trial, highlighting differences in Test and Control (species level, Bray-Curtis dissimilarity on relative abundance). Lines connect different time points contributed from the same subject.


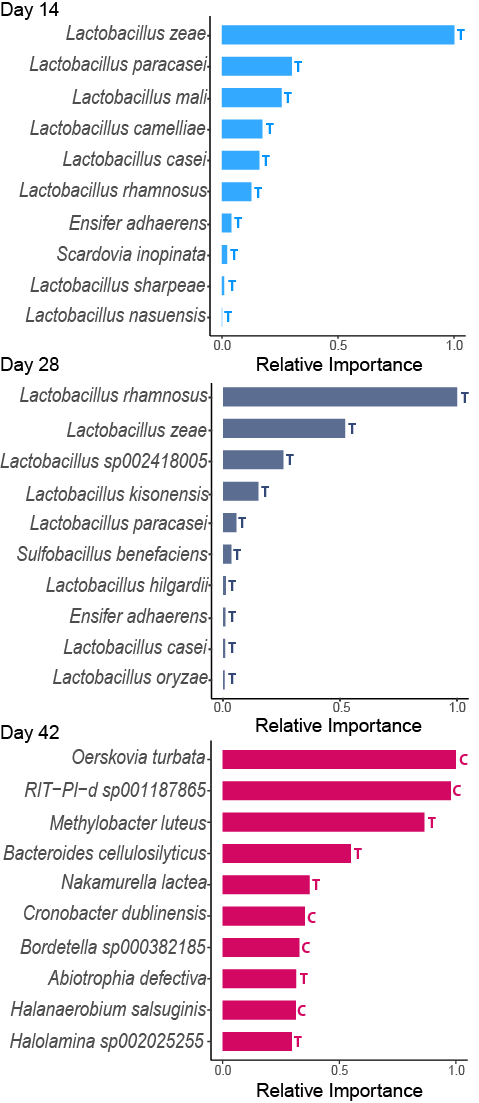


**Figure S5. Relative feature importance for group differentiation by XGBoost.** Displayed are the top 10 most important features for the XGBoost classifiers that distinguish between subjects that consumed the test product and the control group for day 14, 28 and 42, respectively. Feature importance was calculated as the average gain multiplied by the frequency of inclusion in a model. The letters C (control) and T (test product) behind the bars indicate in which group the specified taxon is increased. At both of these time points, the ten most discriminatory taxa used to distinguish Test from Control were almost entirely *Lactobacillus* species, classified either as the test product strains (*L. paracasei, L. rhamnosus*), or as less abundant taxa which are closely-related to the product species (*L. casei, L. zeae,* others). Given the large volume of sequence data being classified, the close taxonomic relationship of these rarer *Lactobacillus* to product strains, and their exact co-incidence with product consumption, these non-product lactobacilli are considered to be misassigned reads from product strains, emphasising the central role of product strains in building discriminatory models at these timepoints.


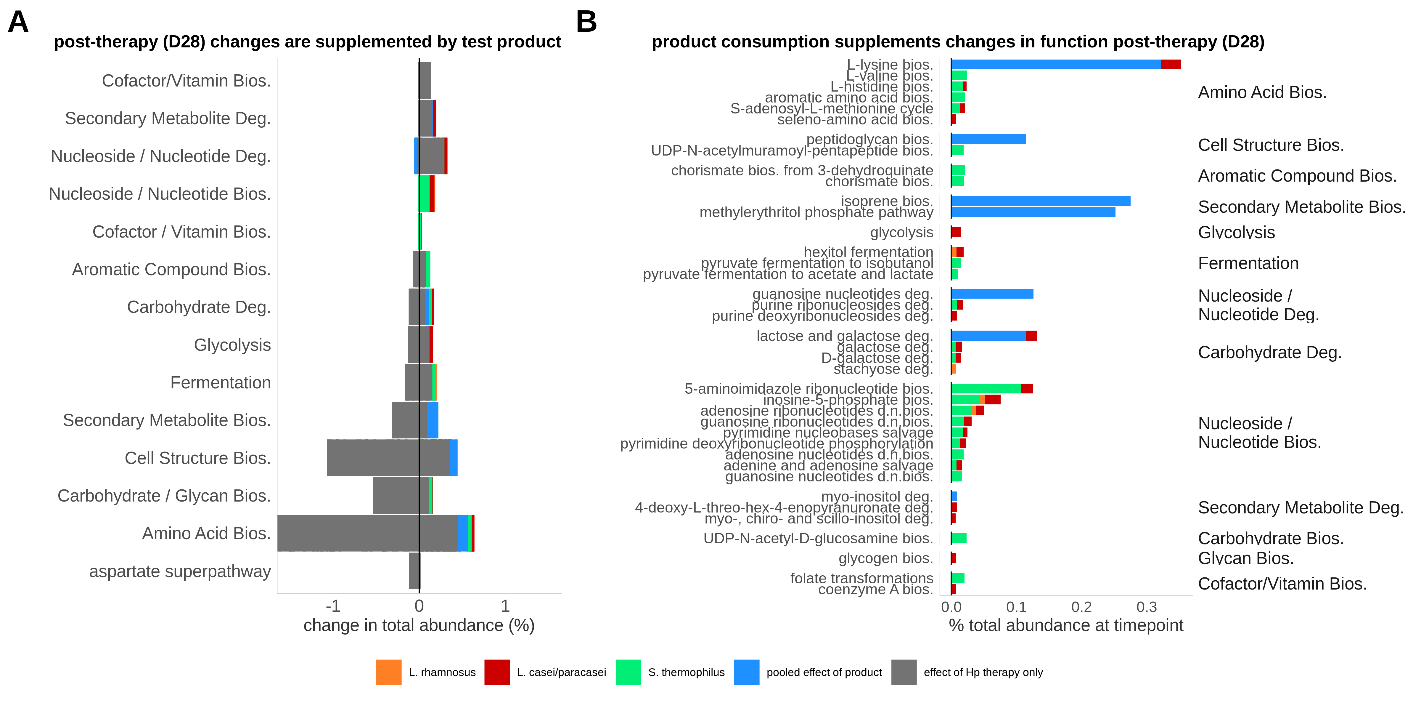


**Figure S6. Pathway relative abundances at D28.** **A.** Grey: overall decreases (left) and increases (right) in total pathway relative abundances due to impact of *Hp* eradication therapy at D28, with reference to D0 (FDR < 0.05); Colored bars show changes significantly associated (FDR < 0.05) with Test product consumption, contributed both directly from the product species (red: *L. paracasei;* orange: *L. rhamnosus*; green: *S. thermophilus*), as well as from the wider gut microbiome (blue: community-level or ‘pooled effect’ of Test product consumption). Apparent decreases in pooled pathway abundance (e.g. nucleoside/nucleotide degradation) represent stochastic differences in starting abundance (D0) between Test and Control, rather than decreases as a result of product consumption. **B.** Significant differences in total functional pathway abundance associated with Test product species at timepoints D28 (red: *L. paracasei;* orange: *L. rhamnosus*; green: *S. thermophilus;* blue: community-level or ‘pooled effect’ of Test product consumption).


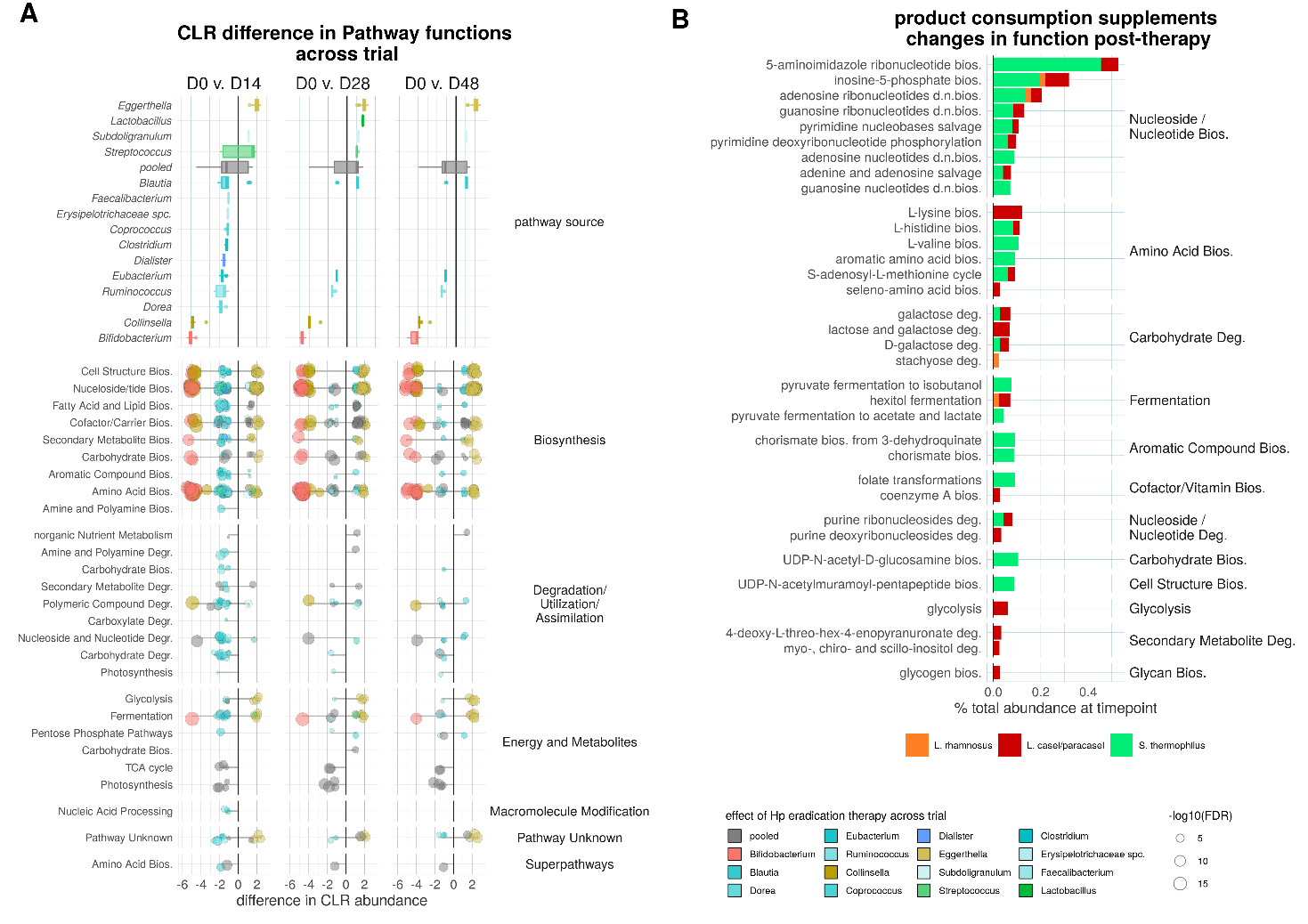


**Figure S7. Differentially abundant functional pathways during Hp eradication therapy and subsequent recovery. A** Differentially abundant pathways, at D0:D14, D0:D28, or D0:D42 in control group. X axis reflects changes in abundance (CLR) from baseline, while size of point reflects -log10 FDR score. Positive difference in CLR indicates higher abundance at the given time point (D14, D28, D42) versus baseline. **B** Significant differences in total functional pathway abundance associated with Test product species at timepoints D14 and D28 (*L. paracasei*: red*; L. rhamnosus*: orange; *S. thermophilus*: green).


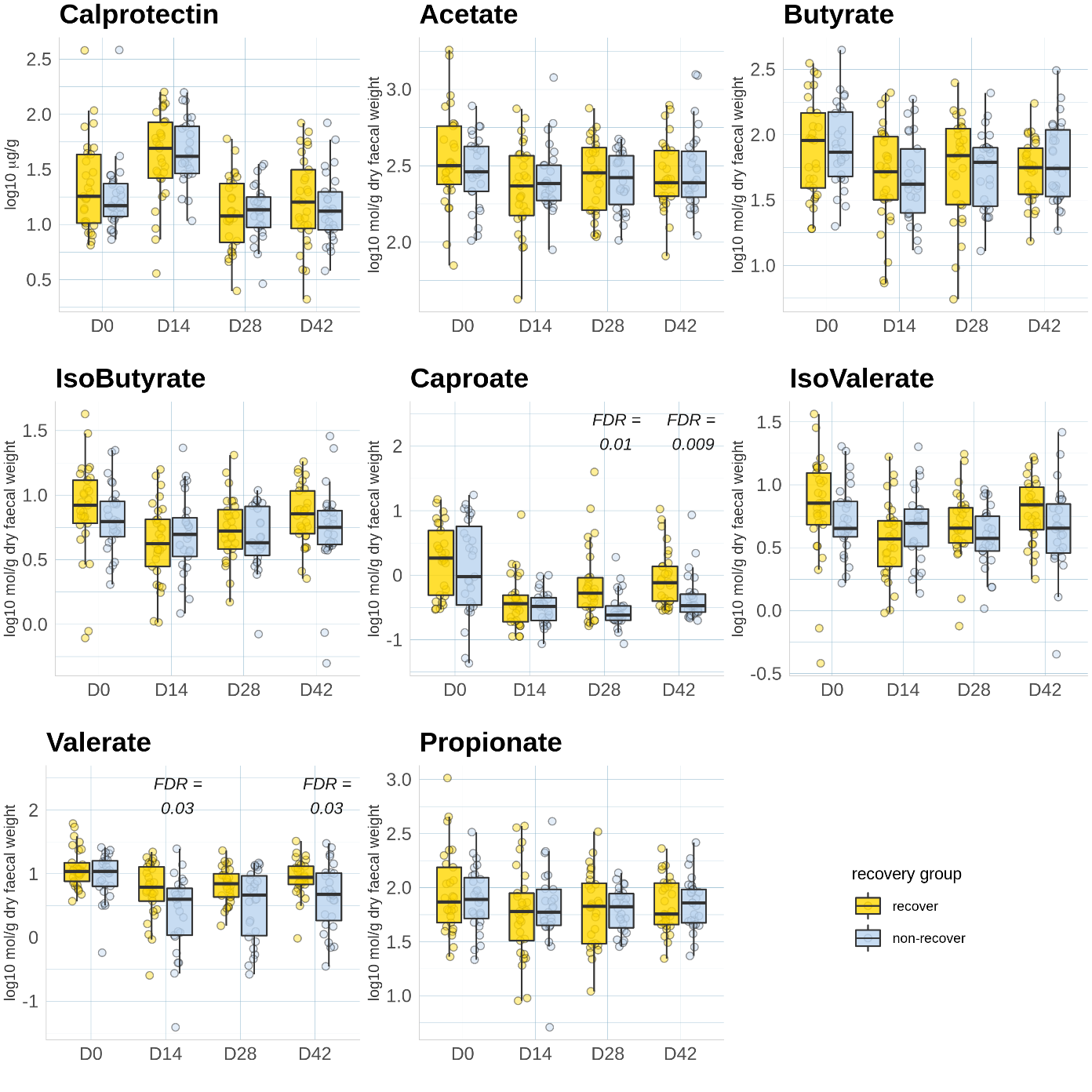


**Figure S8.** Concentrations of calprotectin, short-chain fatty acids (acetate, propionate, butyrate valerate, caproate), branched chain fatty acids (isobutyrate, isovalerate) in recovery and non-recovery groups.

**Supplementary Tables**

**Table S2, S4, S5, S6, S7 and S8 are provided online as excel files**

**Table S1.**  Subject characteristics at baseline

**Table S2.** Strain-based Metagenomic Estimation of Growth (SMEG) scores generated for product strains across trial, and differences between treatment groups related to Figure 2C (Wilcoxon rank-sum, FDR < 0.05)

**Table S3.** Antimicrobial susceptibility of six strains from the fermented milk product using microdilution

**Table S4.** Alpha and beta-diversity statistical tests for species, function and recovery/non-recovery

**Table S5.** Differential analysis for species between baseline and following time points using linear mixed-effect models test (FDR 0.05, |CLR difference| > 1) for control subjects (effect of therapy), between treatment groups (effect of product), and between treatments for co-abundance analysis groups.

**Table S6.** Differential analysis for metabolic pathways between baseline and following time points using linear mixed-effect models (FDR < 0.05, | CLR difference | > 1) for control subjects (effect of therapy) and between groups.

**Table S7.** Differential analysis for species between “recovery” and “non-recovery” between D0 and D42 timepoint, using linear models (FDR < 0.05, | CLR difference | > 1)**.**

**Table S8.** Resource Accessions (NCBI) used to construct databases for Strain-based Metagenomic Estimation of Growth (SMEG)

**Table S1. Subject characteristics at baseline**

|  |  | **Test (N=68)** | **Control (N=68)** |
| --- | --- | --- | --- |
| **Age (years)^1^** | Mean (SD) | 42.1 (10.1) | 42.6 (11.3) |
|  | Min ; Max | 26 ; 65 | 23 ; 64 |
| **Sex, n (%)^1^** | Male | 34 (50.0) | 35 (51.5) |
|  | Female | 34 (50.0) | 33 (48.5) |
| **BMI (kg/m²)^1^** | Mean (SD) | 24.8 (2.9) | 25.0 (2.6) |
|  | Min ; Max | 19.6 ; 30.0 | 19.7 ; 29.5 |
| **Physical activity IPAQ^2^, n (%)^1,^** | Low | 8 (11.8) | 8 (11.8) |
|  | Moderate | 24 (35.3) | 22 (32.4) |
|  | High | 36 (52.9) | 38 (55.9) |
| **Water-insoluble fibers intake (g/day)^1^** | Mean (SD) | 12.96 (4.81) | 11.66 (4.78) |
|  | Min ; Max | 5.91 ; 29.42 | 5.23 ; 28.12 |
| **Water-soluble fibers intake (g/day)^1^** | Mean (SD) | 6.28 (2.60) | 5.75 (2.54) |
|  | Min ; Max | 2.53 ; 15.25 | 2.46 ; 15.5 |
| **Alcohol Consumer, n (%)^1^** |  | 51 (75.0) | 44 (64.7) |
| **Smokers, n (%)^1^** |  | 19 (27.9) | 14 (20.6) |
| ***Hp* eradication rate,n (%)^3^** |  | 57 (83.8) | 60 (88.2) |

^1^ : At baseline (D0)

^2 :^ IPAQ = International Physical Activity Questionnaire

^3 :^ At D42

**Table S3. Antimicrobial susceptibility of six strains from the fermented milk product using microdilution.** According to EUCAST (https://www.eucast.org/), the strains are considered as susceptible to both antibiotics

| Strains | Minimal inhibitory concentrations (mg/l) | |
| --- | --- | --- |
|  | Amoxicillin | Clarithromycin |
| *L. paracasei* CNCM I-1518 | 0.5 | 0.25 |
| *L. paracasei* CNCM I-3689 | 0.5 | 0.25 |
| *L. rhamnosus* CNCM I-3690 | 0.5-1 | 0.25 |
| *S. thermophilus* CNCM I-2773 | 0.06 | 0.25 |
| *S. thermophilus* CNCM I-2835 | 0.12 | 0.25 |
| *S. thermophilus* CNCM I-2778 | 0.12 | 0.12 |
